# The neuropeptide oxytocin modulates consumer brand relationships

**DOI:** 10.1038/srep14960

**Published:** 2015-10-09

**Authors:** Andreas Fürst, Jesko Thron, Dirk Scheele, Nina Marsh, René Hurlemann

**Affiliations:** 1 Department of Marketing, Friedrich-Alexander-University Erlangen-Nürnberg (FAU), 90403 Nürnberg, Germany and Professorial Fellow of the Business School at the University of Eastern Finland (UEF), 70211 Kuopio, Finland; 2 Department of Psychiatry, University of Bonn, 53105 Bonn, Germany; 3 Department of Medical Psychology, University of Bonn, 53105 Bonn, Germany

## Abstract

Each year, companies invest billions of dollars into marketing activities to embellish brands as valuable relationship partners assuming that consumer brand relationships (CBRs) and interpersonal relationships rest upon the same neurobiological underpinnings. Given the crucial role of the neuropeptide oxytocin (OXT) in social bonding, this study tests whether OXT-based mechanisms also determine the bond between consumers and brands. We conducted a randomized, placebo-controlled study involving 101 subjects and analyzed the effect of intranasal OXT on consumers’ attribution of relationship qualities to brands, brands paired with human celebrity endorsers, and familiar persons. OXT indeed promoted the attribution of relationship qualities not only in the case of social and semi-social stimuli, but also brands. Intriguingly, for subjects scoring high on autistic-like traits, the effect of OXT was completely reversed, evident in even lower relationship qualities across all stimulus categories. The importance of OXT in a CBR context is further corroborated by a three-fold increase in endogenous release of OXT following exposure to one’s favorite brand and positive associations between baseline peripheral OXT concentrations and brand relationship qualities. Collectively, our findings indicate that OXT not only plays a fundamental role in developing interpersonal relationships, but also enables relationship formation with objects such as brands.

In modern economics, consumers are faced with a plethora of brands, leaving them with countless choices. This has led to a fundamental paradigm shift in business, replacing a former focus on short-term exchange with a goal of creating reliable and ongoing consumer brand relationships (CBRs)^1^. According to the CBR concept, however, such close ties are only possible when it is assumed that brands are no longer considered as objects, but in fact as social entities^2^,^3^. In line with this, a central prerequisite for such social bonding with brands would therefore require that humans be able to attribute social qualities, such as thoughts and intentions, to non-social objects^4^. In human evolution, tendencies of social attribution may have evolved as a byproduct of the unusually large “social” human brain, which may be necessary to cope with the computational demands of social group-living^5^. By implicitly drawing upon this evolutionarily formed predisposition, companies endeavor to create brands that are perceived as preferable partners for relationships, comparable to friendships, affairs, or even romances. In fact, the successful establishment of CBRs has become increasingly important for practical business success^6^,^7^ and numerous marketing constructs have emerged in line with the assumed social qualities of brands (e.g. brand identification^8^, brand attachment^9^,^10^ or even brand love^11^,^12^). However, since prior research in this field has been mostly qualitative^2^,^13^, surprisingly little is known about whether CBRs are truly comparable to human social bonds in the sense that both engage similar neurobiological underpinnings that determine their existence^14^. Besides providing possible scientific validation of the theoretical CBR concept, neurobiologically informed marketing strategies would also help companies devise specific branding activities that better serve consumer needs while avoiding potential pitfalls.

Drawing upon neurobiological research in the domains of social cognition and behavior, the hypothalamic peptide oxytocin (OXT) has been identified as a key molecule^15^ that mediates social motivation and approach^16^,^17^, fear conditioning and extinction^18^,^19^, social learning and attachment^20^,^21^, pair-bond formation^22^,^23^,^24^, parental care^25^,^26^, and interpersonal trust^27^. An influential concept of OXT’s neuromodulatory effects assumes that the peptide enhances the sensitivity to socially salient cues^28^, possibly by improving the signal-to-noise ratio during neural processing^29^. As such, OXT facilitates the identification^30^ and recognition^31^,^32^ of social stimuli^33^, enhances the reward value of an affiliated partner^34^, and establishes parent-infant relations^35^. However, OXT’s role for the formation, and retention, of CBRs has not been established yet.

We thus hypothesize (*i*) that brands, as a form of non-social stimuli or objects, can be perceived as valuable relationship partners, as are genuine social stimuli (i.e., human). Specifically, as a common neurobiological underpinning, we expect OXT to enable the attribution of relationship qualities to both humans and brands. While actual non-social brands could thus also acquire intrinsic social skills, OXT would also lay the foundation for the emergence of close CBRs. In addition, in light of previous findings, according to which the effects of OXT are particularly strong in case of stimuli to which an individual is already attached^34^, we further predict (*ii*) that the effects of OXT on the attribution of relationship qualities increase with the individual’s attachment to the stimulus. Moreover, previous findings suggest that effects of OXT might depend not only on attachment intensity, but also on interindividual characteristics such as autistic-like personality traits^36^,^37^. Revealing such moderating influences would have considerable implications for companies’ prospective design and management of CBR-promoting activities. Autistic-like personality traits can be measured with a brief 50-item questionnaire, the Autism-spectrum Quotient (AQ)^38^. They are normally distributed in the general population, with the etiologically-linked autism-spectrum disorder representing only the extreme high end of the distribution^39^,^40^. Since the theory of mindblindness^41^ suggests an association between autistic traits and difficulties with social interaction, our third and last hypothesis *(iii)* predicts that the AQ will moderate OXT’s effects on the attribution of relationship qualities. Specifically, we assume that the influence of OXT is positive for individuals with a low AQ, whereas it is diminishing or even detrimental for individuals exhibiting a higher AQ.

To address our hypotheses, we conducted a double-blind, parallel-group pharmacologic challenge experiment in which 101 heterosexually pair-bonded female (*n* = 57) and male (*n* = 44) subjects (mean age ± SD: 24.8 ± 5.2 years) received either a synthetic dose of intranasal OXT (24 IU) or placebo (PLC). Approximately 45 minutes after the administration, participants were asked to rate the relationship qualities of stimuli from three different categories: non-social stimuli (i.e., brands), semi-social stimuli (i.e., brands paired with human celebrity endorsers), and social stimuli (i.e., humans). Furthermore, we also examined whether exposure to these three stimulus categories altered endogenous OXT levels in the PLC group. To this end, we collected saliva samples of each participant before and after the administration of the nasal spray as well as immediately after answering the last item of each stimulus category. In an additional sample of 176 healthy female (*n* = 109) and male (*n* = 67) subjects (20.76 ± 3.10 years), we also measured salivary OXT concentrations and asked the subjects to choose a brand with high attachment and rate the relationship qualities of this brand.

The investigation of social and semi-social stimuli aside from non-social stimuli helps achieve two goals. First, by including social stimuli, we are able to verify the general relevance of OXT as it relates to social stimuli. Second, by implementing semi-social stimuli, we are able to control for the possibility that the attribution of relationship qualities in the case of CBRs may rely on the concomitant presentation of a related and genuinely social stimulus (e.g., celebrity endorser). Moreover, to test our second hypothesis (*ii*), each stimulus category is represented by two stimuli that vary with participants’ a-priori “stimulus attachment”. Participants were therefore asked before the experiment to choose a brand and a celebrity with high and low attachment. With respect to the social stimuli category, participants were asked to name their closest friend or a loose contact representing low and high attachment (for more information regarding implementation and selection of stimuli please see *Materials and methods)*.

Relationship qualities as the dependent variables were operationalized based on the seminal CBR model proposed by Susan Fournier in 1998^2^. Thus, for all stimuli, participants had to rate the perceived *commitment*, *intimacy*, *satisfaction*, *self-connection, trust,* and *loyalty* within the specific relationships. Each construct was measured with three to six items and participants gave their answers on visual analog scales reaching from “strongly disagree” (0) to “strongly agree” (100). By including these subfactors, we wanted to make sure that all facets of the construct “relationship quality” were assessed. We report the results for the global relationship quality index averaging all subfactors in the main text. The results for the subscales and further details regarding construct definitions, scale items, and sources are reported in the Supplemental Information.

## Results

There were no pre-treatment differences between the OXT and PLC group regarding demographics, autism-spectrum quotient (AQ), attachment to stimulus, sex (see Table 1), or neuropsychological performance (see Supplemental Table 1). “Treatment” groups resulted from the random assignment of subjects to intranasal administration of either OXT or PLC nasal spray and “AQ” subgroups were defined by a median split dichotomization (*median* = 14). This procedure yielded a low (*n* = 51; mean AQ ± SD: 10.27 ± 2.52) and a high AQ group (*n* = 50; mean AQ ± SD: 19.62 ± 6.09). Of note, we have only included healthy participants and even the high AQ group exhibited substantially lower AQ scores than patients with autism spectrum disorder (35.8 ± 6.5) in previous studies^38^.

With reference to our first hypothesis (*i*), our following analyses generally aim to verify whether OXT mediates the attribution of relationship qualities to stimuli of three different stimulus categories (i.e., non-social, semi-social, and social). Since hypotheses *ii* and *iii* predict that general treatment effects may be restricted in relation to participants’ “attachment to stimulus” and “AQ”, we tested our hypotheses in reversed order, which allows taking account of potential moderating effects. We initially carried out a repeated-measures analysis of variance (ANOVA) with “treatment” (OXT, PLC) and “AQ” (low, high) as between-subjects factors, “stimulus category” (non-social, semi-social, social) and “attachment to stimulus” (low, high) as within-subjects factors, and the relationship quality index as dependent variable. This analysis yielded highly significant main effects of “stimulus category” (*F*_(1.19,115.21)_ = 288.49, *P* < .01, η^2^ = .75) and “attachment to stimulus” (*F*_(1,97)_ = 1291.45, *P* < .01, η^2^ = .93) as well as a significant interaction of “treatment” and “AQ” (*F*_(1,97)_ = 10.85, *P* < .01, η^2^ = .10). Social stimuli generally received the highest ratings in absolute terms, followed by semi-social and non-social stimuli. Likewise, participants assigned higher ratings to stimuli with high attachment compared to stimuli with low attachment (see Table 2). Further analyses revealed no general influence of sex, age, or years of education.

**Figure 1:**
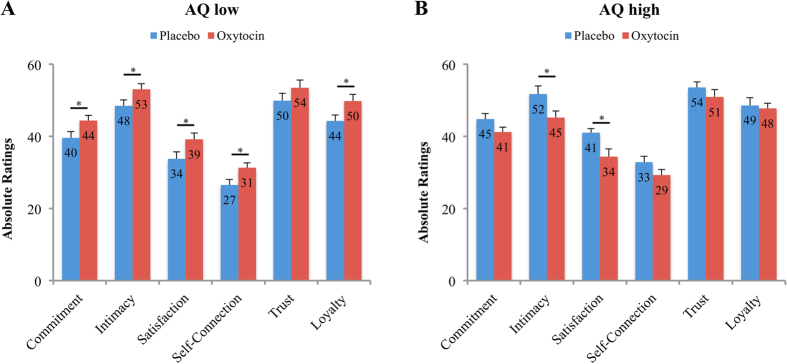
Relationship quality ratings of participants with low and high autistic-like traits under the influence of oxytocin (OXT) and placebo (PLC). Across all stimulus categories the intranasal administration of OXT influenced ratings of relationship qualities. OXT enhanced relationship quality ratings in participants with low autistic traits (**A**), but diminished them in participants with high autistic-like traits (**B**). Error bars indicate the standard error of the mean (SEM). Abbreviations: AQ, autism-spectrum quotient; *P < .05.

**Figure 2:**
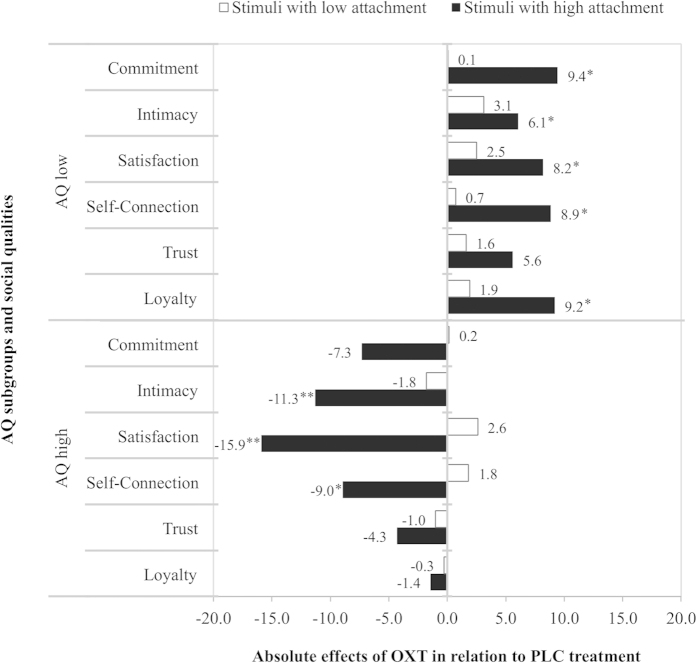
Absolute effects of oxytocin (OXT) in relation to placebo (PLC) on stimuli with low versus high attachment. Across all stimulus categories, the absolute effects of intranasal OXT were more pronounced for stimuli with high a-priori attachment compared to low attachment. Values represent OXT minus PLC ratings. Abbreviations: AQ, autism-spectrum quotient; OXT, oxytocin; PLC, placebo; */**P < .05/.01.

Following the highly significant interaction effects of “treatment” and “AQ”, we next analyzed the general impact of our treatment separately for subjects with AQ low versus high. A repeated-measures ANOVA with the within-subject factors “stimulus category” (non-social, semi-social, social) and “attachment to stimulus” (low, high) as well as the between-subject variable “treatment” (OXT, PLC) revealed the expected opposing effects for the two groups (see Fig. 1). Consistent with our third hypothesis *(iii),* OXT increased relationship quality ratings in the subgroup with AQ low (see Fig. 1A; *F*_(1,49)_ = 6.21, *P* = .02, η^2^ = .11) and reduced them in the subgroup with AQ high (see Fig. 1B; *F*_(1,48)_ = 4.67, *P* = .04, η^2^ = .09) across all stimulus categories. These results indicate that elevated OXT levels made participants with AQ low feel more committed, intimate, satisfied, self-connected, trusting, and loyal towards stimuli, whereas subjects with AQ high revealed opposite effects. However, in line with our second hypothesis *(ii)*, results also show that these effects are particularly pronounced for stimuli with high a-priori attachment as evidenced by interaction effects of “treatment” and “attachment to stimulus” in the AQ low subgroup (*F*_(1,49)_ = 3.08, *P* = .09, η^2^ = .06) and AQ high subgroup (*F*_(1,48)_ = 4.37, *P* = .04, η^2^ = .08). These interaction effects were decomposed by conducting 2 (treatment) x 3 (stimulus category) repeated-measures ANOVAs separately for the stimuli with low and high attachment. Consistent with our second hypothesis (*ii*), OXT effects were evident only for high attachment stimuli, in both subgroups with low AQ scores (*F*_(1,49)_ = 7.03, *P* = .01, η^2^ = .13) and high AQ scores (*F*_(1,48)_ = 6.89, *P* = .01, η^2^ = .13) (see Fig. 2).

However, in addition to these main treatment effects, we again observed significant interactions of “treatment” and “stimulus category” for participants with AQ high (*F*_(1.38,66.41)_ = 10.81, *P* < .01, η^2^ = .18). Consequently, we conducted post hoc *t* tests to examine OXT effects separately for each stimulus category. Table 3 shows that OXT significantly influenced the attribution of relationship qualities under every condition, except for social stimuli and participants with AQ high. More specifically, OXT generally led to increased positive ratings of relationship qualities for participants with AQ low, whereas participants with AQ high tended to assess stimuli’s relationship qualities as rather negative.

**Figure 3:**
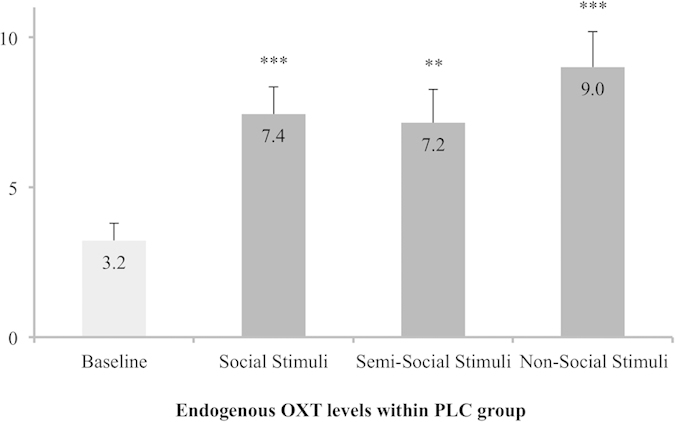
The influence of stimulus categories on endogenous oxytocin (OXT) levels (pg/ml) in the placebo (PLC) group. In the PLC group, the presentation and rating of social, semi-social, and non-social stimuli induced a significant release of endogenous OXT compared to the baseline measurement before the start of the experiment. Importantly, the strongest increment in OXT levels was evident in the non-social (i.e., brand) condition. Error bars indicate the standard error of the mean (SEM). Abbreviations: OXT, oxytocin; PLC, placebo; **/***P < .01/.001.

Further support for the notion that the bonding with brands and genuinely social stimuli share common neurobiological underpinnings comes from our analysis of endogenous OXT levels within the PLC group. Showing no differences between the AQ low and AQ high subgroup, baseline levels of salivary OXT were more than doubled after evaluation of social (*t*_(46)_ = 4.77, *P* < .001, η^2^ = .33) and semi-social stimuli (*t*_(47)_ = 3.30, *P* =  < .01, η^2^ = .19). Importantly, the increment of OXT levels was even stronger (i.e., almost tripled) following the presentation of non-social stimuli (*t*_(47)_ = 4.80, *P* = < .001, η^2^ = .33, see Fig. 3), thus indicating that dealing with attached objects (i.e., selected brands) can induce an endogenous OXT release that is even more pronounced than that caused by interactions with human beings.

**Figure 4:**
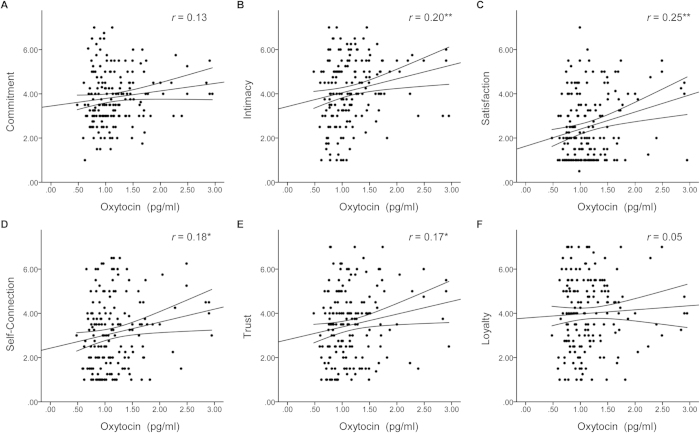
Associations between endogenous oxytocin (OXT) levels and relationship quality ratings. In an additional sample of 176 healthy participants, the correlation between salivary OXT concentrations and commitment (**A**), intimacy (**B**), satisfaction (**C**), self-connection (**D**), trust (**E**), and loyalty (**F**) ratings of brands with high attachment were examined. Weak, but significant positive associations were evident for intimacy, satisfaction, self-connection, and trust. The curves next to the regression lines indicate 95% confidence intervals. Abbreviations: */**P < .05/.01.

In the additional sample of 176 participants, we observed no association between salivary OXT concentrations and autistic-like traits (*r* = −0.01, *P* = .92). However, we detected weak but significant correlations between salivary OXT concentrations and the relationship quality index (*r* = 0.21, *P* < .01). In the analysis with the relationship subscales, significant associations were evident for ratings of brand intimacy (*r* = 0.20, *P* < .01), satisfaction (*r* = 0.25, *P* < .01), self-connection (*r* = 0.18, *P* = .02), and trust (*r* = 0.17, *P* = .03) (see Fig. 4A–F).

## Discussion

In the present study, we aimed to test the notion that consumer-to-brand relationships are constituted by the same neurobiological substrates as interpersonal ones. Consistent with this idea, we found that the neuropeptide OXT significantly influenced the relationship quality ratings of non-social (i.e., mere brands), semi-social (i.e., brands paired with human celebrity endorsers), and truly social stimuli (e.g. friends or neighbors). Notably, the mediating influence of intranasal OXT administration and the increase in endogenous OXT levels were even stronger for non-social, compared to semi-social, stimuli, suggesting that brands do not require the simultaneous presentation of a genuinely social stimulus (e.g., human celebrity endorser) to be processed as social relationship partners. Our study points to a couple of specific implications that seem valuable from both a practical and theoretical point of view.

**Figure 5:**
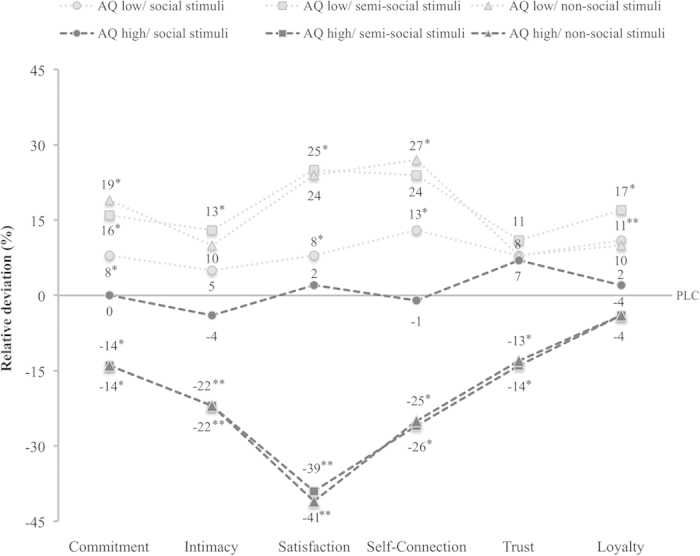
Relative effects of oxytocin (OXT) on relationship quality ratings for different stimulus categories. Relative treatment effects (OXT minus placebo in %) on relationship quality ratings of stimuli with high attachment were particularly pronounced for semi- and non-social stimuli. While relationship quality ratings were generally increased in the low and diminished in the AQ high subgroup, the strongest relative effects were observed for ‘satisfaction’ and ‘self-connection’ ratings of non-social stimuli. Abbreviations: AQ, autism-spectrum quotient; OXT, oxytocin; PLC, placebo; */**P < .05/.01.

First, the administration of a single dose of synthetic OXT influenced the attribution of several specific qualities within CBRs, but the effects were particularly strong and most diverse in the low versus high AQ subgroups for the satisfaction with the relationship partner (see Fig. 5). Since satisfaction is usually associated with a pleasant or even rewarding feeling, this pattern of results resonates well with our previous finding that OXT enhances neural responses in brain reward regions if men were presented with photographs of their female partners’ faces as compared to unfamiliar women^34^. However, because satisfaction has been identified as a primary predictor of consumers’ future intentions^42^, this finding is even more important in the context of our study’s specific CBR operationalization. While previous studies mainly emphasized the relevance of brand “familiarity” as a key factor in the perception of a brand as satisfying or rewarding^43^,^44^, our results rather suggest that OXT-mediated forms of CBRs might establish both brand familiarity and subsequent feelings of reward. However, further research is needed to conclusively determine fundamental processes and possible interactions with other neurotransmitter systems (e.g. dopamine or serotonin) that mediate the rewarding nature of brands. Considering that a familiar brand is not necessarily a rewarding brand, new research should also clarify the antecedents and external conditions that enable this transformation.

In terms of the practical economic point of view, the specific implications of relationship qualities being attributed to a non-social brand proved far-reaching and double-edged. As shown, the attribution of relationship qualities to a brand can indeed have invigorating effects in some individuals, but also detrimental effects in others, depending on their AQ. Specifically, while intranasal OXT in combination with AQ low led to a strong increase in the relationship commitment, intimacy, satisfaction, self-connection, trust, and loyalty, the exact opposite effect occurred in combination with AQ high. Consequently, since the distribution of autistic-like traits within our sample (14.9 ± 6.6) was a fair but modest representation of the population in general (16.4 ± 6.3)^38^, managers’ common attempts to grace brands with an appealing “human touch” irrespective of their target groups’ main personality traits are tenuous at best. While the strategy of “socializing” brands appears to be particularly expedient for those that are rather “impersonal” by nature (e.g., due to a technical or financial product benefit), disastrously, average AQ scores may be highest for members of corresponding target groups. This may be due to the fact that autistic traits vary as a function of individuals’ affinity for science, figures, or technology, with mathematicians, physicists, and computer scientists typically displaying higher scores than, for instance, humanities scholars^38^. One possible explanation for the contrary effects of OXT between the AQ subgroups could be related to differing feelings experienced by AQ low/AQ high subjects when they are presented with an inanimate, yet “social” object. While subjects with a high AQ might feel constrained or even trapped when a critical social boundary is exceeded, low AQ subjects might positively relate to a stronger feeling of pleasure and affection. Such effects correspond with findings that suggest a range of autism-related social impairments, such that some people react to social interaction in a passive and withdrawing manner, while others actively engage in interactions in an odd, even bizarre, way^45^. Although we did not measure tendencies for social attribution directly, it is also conceivable that OXT may have enhanced the attribution of social meaning, which is usually diminished in individuals with high autistic traits^46^,^47^. In fact, we have recently shown that OXT promotes sponatenous anthropomorphism in women^48^. The devaluation of a brand’s relationship qualities may thus be the consequence of an artificially augmented unfamiliar tendency of social attribution. Mechanistically, OXT may have also altered empathic accuracy and attachment styles^36^,^49^,^50^ which have been proposed as avenues through which individual differences can systematically affect brand perceptions^51^.

Returning to findings that are generally relevant in the context of human-to-object relationships, we extend previous reports of an OXT-induced bias for the overall liking of in-group cultural stimuli^52^ by demonstrating that the modulatory effect of OXT is not restricted to specific stimulus categories, but may rather depend on a-priori attachment. Furthermore, several studies have documented the relevance of peripheral OXT concentrations as biomarkers for affiliative behavior and bonding. For instance, elevated peripheral levels of OXT have been found after a 15 minute play-and-contact interaction between parents and infants^53^, warm partner contact^54^, a massage^55^ or sexual intercourse^56^. In the present study, the presentation of both social stimuli and non-social stimuli (i.e., mere brands) evoked a release of endogenous OXT, thus indicating that high attachment stimuli, even in the absence of genuinely social elements, are sufficient to stimulate the OXT system. Interestingly, the increase in OXT levels was evident across AQ subgroups, which may suggest that CBRs in AQ high scorers are also mediated by oxytocinergic mechanisms, but an additional augmentation of relationship-related stimulus features by exogenous OXT produces detrimental effects in this population. Furthermore, our observation of weak but significant associations between baseline salivary OXT concentrations and brand relationship qualities also support the idea that CBRs rest upon the OXT system. Again, the strongest effect was evident for satisfaction ratings. Of note, the effect sizes of the observed correlations were small in all conditions. Given the still unclear relationship between peripheral and central OXT measurements^57^,^58^, future studies are warranted to establish the functional relevance of this finding in the brain.

In sum, we provide the first scientific evidence that there is an overlap in the neurobiological substrates mediating the attribution of relationship qualities to social and non-social objects such as brands. In doing so, our study offers valuable support for the core assumptions of economical CBR models and related marketing strategies^2^,^3^,^6^,^9^. By showing that brands with high attachment can engage the OXT system similarly to real humans, we reveal that individuals are able to form relationships with their favorite brands that, in many ways, resemble interpersonal ones. Importantly, this effect does not require the presence of supporting social cues such as celebrity endorsers. While this insight calls for managerial efforts to establish and maintain CBRs, our observation that the modulatory influence of intranasal OXT is dependent upon interindividual differences in autistic-like traits strictly indicates that corresponding campaigns should be individually tailored to the personality characteristics of different target groups.

## Materials and Methods

### Participants

One hundred and one healthy, nonsmoking female (*n* = 57) and male (*n* = 44) adults participated in the intranasal OXT study after giving written informed consent. Due to the exploratory character of this study and the lack of studies investigating a comparable question, we abstained from specific a-priori power calculations of the necessary sample size. Given the moderate effect sizes obtained in previous behavioral OXT studies^59^, we planned to recruit at least 25 participants for each of the four conditions (OXT, PLC, AQ high, and AQ low). Subjects received monetary compensation for study participation. Four subjects were excluded from analysis showing scores >10 on the Beck Depressions Inventory (BDI)^60^ as well as abnormalities in the Mini-International Neuropsychiatric Interview (MINI)^61^. Subjects were free of current and past physical or psychiatric illness, as assessed by medical history and the MINI. In addition, they were naive to prescription-strength psychoactive medication and had not taken any over-the-counter psychoactive medication in the preceding four weeks. All subjects were in a romantic heterosexual relationship for more than six months and had no children. For the additional assessment of salivary OXT concentrations, we recruited 176 healthy female (*n* = 109) and male (*n* = 67) subjects. Five participants were excluded from the analysis showing salivary OXT concentrations which deviated more than 2.5 standard deviations from the mean. Both studies were approved by the institutional review board of the Medical Faculty of the University of Bonn and were carried out in compliance with the latest revision of the Declaration of Helsinki.

### Overall procedure

The intranasal OXT study consisted of two separated sessions. In the first session, participants were screened regarding current and past physical or psychiatric illness as well as neuropsychological performance (Table S1). Subsequently, subjects were asked to choose their individual set of stimuli and rate their attachment to each stimulus. Since participants had to select the stimuli during the first session, we made sure no important changes concerning any of the chosen stimuli occurred until the date of the actual experiment. On arrival at the laboratory for the second session, subjects were randomly assigned to either intranasal administration of OXT (24 IU; Syntocinon-Spray, Sigma Tau; three puffs per nostril, each with 4 IU OXT) or PLC (containing identical ingredients as the OXT-Spray except the neuropeptide itself). Before and 45 min after self-administration of the nasal spray, subjects were asked to submit a saliva sample. Subsequently, participants were seated in front of a computer screen and conducted the experimental task, which took another 30 min to complete. During the experiment, three additional saliva samples were collected shortly after the presentation of each stimulus category. Upon finishing the final questionnaire, subjects were thanked and dismissed. Saliva samples in the additional study with 176 healthy participants were collected in one session at 9 am. All saliva samples were collected with commercial sampling devices (Salivettes, Sarstedt) and Salivettes were immediately centrifuged at 4,180 g for 2 min and aliquoted samples were stored at −80 °C until assayed. Saliva OXT was extracted and quantified by a highly sensitive and specific radioimmunoassay (RIAgnosis, Munich, Germany)^62^. The limit of detection was 0.1–0.5 pg depending on the age of the tracer. Intra-assay and inter-assay coefficients of variability were <10%. All samples to be compared were assayed in the same batch, i.e. under intra-assay conditions.

### Stimuli

In the intranasal OXT study, each stimulus category (non-social, semi-social, and social) contained stimuli with low and high attachment. For the social stimulus category, participants were asked to name their closest friend (high attachment) as well as another familiar, but not close, person (low attachment). Regarding non-social stimuli, subjects were provided with a shortlist of 20 different brands and were asked to pick one brand with high, and one with low, attachment. All brands belonged to the category of food and beverages as well as body care products. Brand relationship theory^63^ covers all product categories, but we opted for this set compilation based on the availability of testimonials and to ensure the comparability in relation to a common superior product category (i.e., “fast-moving consumer goods”). For semi-social stimuli participants were asked to add one celebrity endorser to their previously chosen brands that, in the first case, increased (high attachment semi-social stimulus) and, in the second case, further decreased (low attachment semi-social stimulus) their attachment to the respective brands. Furthermore, all participants were asked to rate their attachment to each stimulus with regard to the following definition of attachment on a seven point Likert scale: “Attachment can be understood as a perceived connectedness or belongingness between the self and another subject or object, which is reflected by positive thoughts, associations and emotional feelings that come to mind intuitively when being exposed to that specific attachment subject or object”^9^,^64^. Non-social stimuli consisted of the brand name and logo, while semi-social stimuli comprised the brand name and photograph of the chosen celebrity endorser. In the social category, the photograph and name of the close friend were presented in high attachment trials and the name and schematic illustration of the familiar, but not close, person were shown in low attachment trials. To avoid confounding factors in the photographs, the backgrounds were masked in gray. In the additional study, the 176 participants were provided with the same shortlist of 20 different brands as in the intranasal OXT study, but the participants only had to pick one brand with high attachment.

### Study design

In the intranasal OXT study, we applied a placebo-controlled, double-blind, parallel-group design. The factors “stimulus category” (non-social, semi-social, and social) and “attachment to stimulus” (low, high) were used as within-subject variables. The presentation order of the stimulus categories and attachment intensities was randomized with the exception that non-social stimuli always preceded semi-social stimuli. We thus can exclude the possibility that celebrity endorsers exerted any spillover effects on mere brand presentation.

### Experimental task

In the intranasal OXT study, subjects were introduced to the experimental task by the experimenter. After a short training session on the correct usage of the visual analog scales, a total of 26 items representing the different dependent variables had to be assessed for each stimulus. Every item contained the name of the specific stimulus and was complemented by a pictorial illustration presented above (for specific items and sources please see Table S2). Participants had no time restrictions and were offered a self-determined break after the presentation of each stimulus category. Items used for the relationship quality ratings in the additional study with peripheral measurements are indicated in Table S2. A reliability analysis revealed an acceptable internal consistency (all Cronbach’s Alphas >0.66) between the relationship quality subscales in all conditions (i.e. “stimulus category” (non-social, semi-social, and social) and “attachment to stimulus” (low, high)).

## Additional Information

**How to cite this article**: Fürst, A. *et al.* The neuropeptide oxytocin modulates consumer brand relationships. *Sci. Rep.* **5**, 14960; doi: 10.1038/srep14960 (2015).

## Supplementary Material

Supplementary Information

## Figures and Tables

**Table 1:** Demographics, autistic personality traits (AQ), and a-priori attachment to stimulus.

	OXT group	PLC group	*t*	*P*
	Mean (SD)	Mean (SD)		
Age (years)	25.02 (5.48)	24.50 (4.91)	0.51	0.61
Education (years)	16.74 (2.65)	16.21 (2.49)	1.00	0.31
Autism-spectrum Quotient (AQ)	15.22 (6.66)	14.59 (6.58)	0.48	0.63
Non-social stimuli (low attachment^1^)	0.12 (0.22)	0.19 (0.26)	−1.41	0.16
Non-social stimuli (high attachment^1^)	4.35 (1.60)	4.64 (1.56)	−0.93	0.35
Semi-social stimuli (low attachment^1^)	0.11 (0.18)	0.20 (0.32)	−1.64	0.10
Semi-social stimuli (high attachment^1^)	5.05 (1.58)	5.32 (1.25)	−0.97	0.34
Social stimuli (low attachment^1^)	0.59 (0.84)	0.56 (0.58)	0.22	0.83
Social stimuli (high attachment^1^)	5.27 (1.39)	5.16 (1.33)	0.42	0.68
	Absolute	Absolute	***χ^2^***	*P*
Sex (female/male)	29/21	28/23	1.00	0.75

*Notes.*

^1^Baseline attachment to stimuli was assessed using participants’ agreement on a definition of attachment towards the different stimuli (maximum possible score 7). OXT, oxytocin; PLC, placebo.

**Table 2:** Overall relationship quality ratings for different stimulus categories and attachment intensities.

	Mean (SD)					
	Commitment	Intimacy	Satisfaction	Self-Connection	Trust	Loyalty
Non-social stimuli	35.6 (8.9)	41.2 (11.6)	25.1 (13.1)	21.3 (9.2)	45.2 (12.0)	43.5 (11.4)
Semi-social stimuli	38.1 (9.9)	40.9 (11.8)	29.5 (14.0)	25.0 (10.7)	45.6 (11.7)	43.7 (11.4)
Social stimuli	53.2 (11.0)	66.0 (12.3)	55.8 (12.9)	43.1 (10.3)	64.7 (14.2)	55.2 (13.4)
Low attachment stimuli	10.3 (8.6)	27.3 (12.9)	15.4 (11.5)	7.6 (5.6)	35.3 (12.0)	17.8 (11.8)
High attachment stimuli	74.3 (13.9)	71.5 (12.4)	58.3 (15.2)	52.0 (14.8)	68.3 (12.5)	77.1 (13.8)

**Table 3:** Relationship quality ratings for different stimulus categories according to treatment and autistic traits.

	AQ low				AQ high			
	Mean (SD)		*t*	*P*^1^	Mean (SD)		*t*	*P*
	OXT group	PLC group			OXT group	PLC group		
*Non-Social Stimuli*^2^								
Commitment	69.59 (16.65)	58.43 (18.65)	2.23	0.02	61.35 (15.20)	71.61 (14.77)	−2.41	0.02
Intimacy	64.74 (16.26)	58.70 (17.39)	1.27	0.11	55.03 (15.64)	70.13 (16.48)	−3.32	<0.01
Satisfaction	41.93 (16.19)	33.84 (21.77)	1.48	0.07	32.97 (18.58)	55.79 (20.22)	−4.16	<0.01
Self-Connection	40.72 (16.47)	32.05 (16.28)	1.88	0.03	35.13 (17.48)	47.06 (16.18)	−2.49	0.02
Trust	59.50 (14.76)	55.29 (19.24)	0.86	0.20	57.62 (15.28)	66.30 (12.57)	−2.17	0.04
Loyalty	72.48 (17.68)	65.88 (19.70)	1.25	0.11	73.80 (18.04)	77.20 (15.83)	−0.70	0.49
*Semi-Social Stimuli*^2^								
Commitment	75.97 (15.49)	65.30 (23.84)	1.85	0.04	67.82 (17.84)	79.23 (15.06)	−2.42	0.02
Intimacy	67.96 (12.40)	60.25 (17.48)	1.78	0.04	55.37 (19.19)	70.91 (19.35)	−2.84	<0.01
Satisfaction	49.96 (19.95)	39.92 (21.59)	1.71	0.04	40.42 (24.26)	66.65 (18.11)	−4.27	<0.01
Self-Connection	47.56 (18.92)	38.47 (19.89)	1.66	0.05	41.48 (18.91)	55.73 (19.02)	−2.65	0.01
Trust	64.55 (15.23)	58.18 (19.31)	1.29	0.10	60.34 (17.84)	69.80 (13.34)	−2.09	0.04
Loyalty	80.38 (14.51)	68.73 (22.39)	2.15	0.02	75.22 (18.36)	77.98 (15.57)	−0.57	0.57
*Social Stimuli*^2^								
Commitment	91.88 (10.15)	85.42 (14.86)	1.77	0.04	84.65 (12.01)	84.90 (15.86)	−0.06	0.95
Intimacy	93.20 (7.23)	88.76 (11.03)	1.66	0.05	86.89 (9.48)	90.13 (8.29)	−1.28	0.21
Satisfaction	91.70 (8.27)	85.27 (12.92)	2.06	0.02	84.18 (15.02)	82.88 (17.02)	0.29	0.78
Self-Connection	78.37 (15.74)	69.57 (17.18)	1.89	0.03	71.03 (15.67)	71.73 (19.44)	−0.14	0.89
Trust	87.59 (11.53)	81.32 (16.44)	1.60	0.06	83.82 (13.40)	78.59 (16.84)	1.22	0.23
Loyalty	91.23 (8.26)	81.94 (17.03)	2.55	<0.01	82.95 (13.20)	81.07 (17.01)	0.44	0.66

*Notes.*

^1^According to hypothesis *(iii), t* tests for the AQ low subgroup were one-tailed.

^2^Stimuli with high attachment; AQ, autism-spectrum quotient; SD, standard deviation; OXT, oxytocin; PLC, placebo.
