# Supplementary material for: The neuropeptide oxytocin modulates consumer brand relationships
Source: Sci Rep. 2015 Oct 9;5:14960. doi: 10.1038/srep14960 (PMC4598816; doi:10.1038/srep14960)
Supplement: Supplementary Information [file srep14960-s1.doc]

**Supplemental Information:**

**The neuropeptide oxytocin modulates consumer brand relationships**

Andreas Fürst, Jesko Thron, Dirk Scheele, Nina Marsh and René Hurlemann

**Supplemental Results:**

We carried out a repeated-measures analysis of variance (ANOVA) with “treatment” (OXT, PLC) and “AQ” (low, high) as between-subjects factors, “stimulus category” (non-social, semi-social, social) and “attachment to stimulus” (low, high) as within-subjects factors, and ratings of “relationship qualities” (commitment (CO), intimacy (IN), satisfaction (SA), self-connection (SC), trust (TR), loyalty (LO)) as dependent variables. This analysis yielded highly significant main effects of “stimulus category” (CO: *F*_(1.28,123.88)_ = 174.12, *P* < .01, ƞ^2^ = .64; IN: *F*_(1.28,124.47)_ = 284.66, *P* < .01, ƞ^2^ = .75; SA: *F*_(1.38,131.62)_ = 234.23, *P* < .01, ƞ^2^ = .71; SC: *F*_(1.49,144.78)_ = 247.90, *P* < .01, ƞ^2^ = .72; TR: *F*_(1.36,131.91)_ = 132.95, *P* < .01, ƞ^2^ = .58; LO: *F*_(1.36,132.25)_ = 45.05, *P* < .01, ƞ^2^ = .32) and “attachment to stimulus” (CO: *F*_(1,97)_ = 1548.51, *P* < .01, ƞ^2^ = .94; IN: *F*_(1,97)_ = 743.5, *P* < .01, ƞ^2^ = .89; SA: *F*_(1,97)_ = 601.94, *P* < .01, ƞ^2^ = .86; SC: *F*_(1,97)_ = 873.3, *P* < .01, ƞ^2^ = .90; TR: *F*_(1,97)_ = 521.33, *P* < .01, ƞ^2^ = .84; LO: *F*_(1,97)_ = 1056.69, *P* < .01, ƞ^2^ = .92) as well as significant interactions of “treatment” and “AQ” (CO: *F*_(1,97)_ = 7.14, *P* < .01, ƞ^2^ = .07; IN: *F*_(1,97)_ = 9.15, *P* < .01, ƞ^2^ = .09; SA: *F*_(1,97)_ = 10.54, *P* < .01, ƞ^2^ = .10; SC: *F*_(1,97)_ = 7.21, *P* < .01, ƞ^2^ = .07). Social stimuli generally received the highest ratings in absolute terms, followed by semi-social and non-social stimuli. Likewise, participants assigned higher ratings to stimuli with high attachment compared to stimuli with low attachment.

A repeated-measures ANOVA with the within-subject factors “stimulus category” (non-social, semi-social, social) and “attachment to stimulus” (low, high) as well as the between-subject variable “treatment” (OXT, PLC) for the six dependent variables (CO, IN, SA, SC, TR, LO) revealed that OXT increased relationship quality ratings in the subgroup with AQ low (CO: *F*_(1,49)_ = 4.09, *P* = .049, ƞ^2^ = .08; IN: *F*_(1,49)_ = 4.07, *P* = .049, ƞ^2^ = .08; SA: *F*_(1,49)_ = 4.11, *P* = .048, ƞ^2^ = .08; SC: *F*_(1,49)_ = 5.41, *P* = .024, ƞ^2^ = .10; LO: *F*_(1,49)_ = 4.97, *P* = .030, ƞ^2^ = .09) and reduced them in the subgroup with AQ high (IN: *F*_(1,48)_ = 5.09, *P* = .03, ƞ^2^ = .10; SA: *F*_(1,48)_ = 6.62, *P* = .01, ƞ^2^ = .12) across all stimulus categories. These results indicate that elevated OXT levels made participants with AQ low feel more committed, intimate, satisfied, self-connected, trusting, and loyal towards stimuli, whereas subjects with AQ high revealed opposite effects. However, in line with our second hypothesis *(ii)*, results also show that these effects are particularly pronounced for stimuli with high a-priori attachment as evidenced by several significant interaction effects of “treatment” and “attachment to stimulus” in the AQ low subgroup (CO: *F*_(1,49)_ = 4.97, *P* = .03, ƞ^2^ = .09; SC: *F*_(1,49)_ = 3.63, *P* = .06, ƞ^2^ = .07) and AQ high subgroup (IN: *F*_(1,48)_ = 3.79, *P* = .05, ƞ^2^ = .07; SA: *F*_(1,48)_ = 11.09, *P* < .01, ƞ^2^ = .19; SC: *F*_(1,48)_ = 6.27, *P* = .02, ƞ^2^ = .12). These interaction effects were decomposed by conducting a 2 (treatment) x 3 (stimulus category) repeated-measures ANOVA separately for the stimuli with low and high attachment. Consistent with our second hypothesis (*ii*), OXT effects were evident only for high attachment stimuli, in both subgroups with low AQ scores (IN: *F*_(1,48)_ = 9.67, *P* < .01, ƞ^2^ = .17; SA: *F*_(1,48)_ = 14.30, *P* < .01, ƞ^2^ = .23; SC: *F*_(1,48)_ = 4.63, *P* = .04, ƞ^2^ = .09) and high AQ scores (CO: *F*_(1,49)_ = 5.94, *P* = .02, ƞ^2^ = .11; IN: *F*_(1,49)_ = 4.19, *P* = .046, ƞ^2^ = .08; SA: *F*_(1,49)_ = 5.05, *P* = .03, ƞ^2^ = .09; SC: *F*_(1,49)_ = 5.08, *P* = .03, ƞ^2^ = .09; LO: *F*_(1,49)_ = 5.64, *P* = .02, ƞ^2^ = .10). However, in addition to these main treatment effects, we again observed significant interactions of “treatment” and “stimulus category” for participants with AQ high (CO: *F*_(1.48,71.11)_ = 5.10, *P* = .02, ƞ^2^ = .10; IN: *F*_(1.39,66.71)_ = 5.58, *P* = .01, ƞ^2^ = .10; SA: *F*_(1.56,75.06)_ = 12.76, *P* < .01, ƞ^2^ = .21; SC: *F*_(1.45,69.73)_ = 4.27, *P* = .03, ƞ^2^ = .08; TR: *F*_(1.43,68.75)_ = 8.08, *P* < .01, ƞ^2^ = .14).

Importantly, the AQ high and AQ low groups were comparable with regard to neuropsychological performance (all tests reported in Table S1, except the AQ score), baseline salivary OXT levels, and baseline brand attachment (all *P*s > .05).

**Tables:**

| **Table S1:** Neuropsychological performance. | | |  |  |
| --- | --- | --- | --- | --- |
|  | Mean (SD) | | *t* | *P* |
|  | OXT group | PLC group |  |  |
| LPS-4 ^1^ | 31.26 (4.09) | 31.92 (4.17) | -0.81 | .42 |
| MWT-B ^2^ | 29.74 (3.37) | 29.59 (3.71) | 0.22 | .83 |
| d2 ^3^ | 198.12 (50.87) | 198.45 (42.27) | -0.04 | .97 |
| TMT-A ^4^ | 26.08 (9.82) | 22.79 (7.57) | 1.87 | .06 |
| TMT-B ^4^ | 61.98 (20.26) | 57.23 (24.03) | 1.06 | .29 |
| Digit-span, forward ^5^ | 9.02 (2.36) | 8.47 (2.00) | 1.27 | .21 |
| Digit-span, backwards ^5^ | 8.14 (2.15) | 8.24 (2.07) | -0.23 | .82 |
| BDI ^6^ | 3.86 (3.50) | 2.80 (3.95) | 1.42 | .16 |
| AQ ^7^ | 15.22 (6.66) | 14.59 (6.57) | 0.48 | .63 |
| PANAS-PA-Pre ^8^ | 31.70 (5.60) | 31.43 (4.93) | 0.26 | .80 |
| PANAS-NA-Pre ^8^ | 12.00 (2.28) | 11.37 (1.51) | 1.64 | .11 |
| PANAS-PA-Post ^8^ | 31.38 (6.48) | 31.33 (7.11) | 0.34 | .97 |
| PANAS-NA-Post ^8^ | 10.76 (1.67) | 10.55 (1.19) | 0.73 | .47 |
| STAI-X1-Pre ^9^ | 33.76 (4.77) | 33.78 (5.00) | -0.03 | .98 |
| STAI-X1-Post ^9^ | 32.30 (5.06) | 30.84 (5.39) | 1.4 | .17 |
| STAI-X2 ^9^ | 35.06 (7.86) | 32.41 (6.72) | 1.82 | .07 |

*Notes.* ^1^ Nonverbal reasoning IQ was assessed by the LPS (Leistungsprüfsystem) subtest 4 (maximum possible score 40);
^2^ Verbal IQ based on lexical decisions was assessed by the MWT-B (Mehrfachwahl-Wortschatz-Intelligenz-Test Teil B) (maximum possible score 37); ^3^ Visual attention and concentration was assessed using the d2 (Aufmerksamkeits- und Belastungstest d2); ^4^ Visual attention and task-switching was assessed using the TMT-A and TMT-B (Trail-making test A, B) (results displayed in seconds); ^5^ Working memory performance was assessed using the digit-span forward and backward test (maximum possible score 14); ^6^ Depressive symptoms were assessed by the self-report BDI (Beck Depression Inventory, Version II); ^8^ Autistic personality traits were assessed using the AQ (Autism-Spectrum Quotient, maximum possible score 50); ^8^ Affect was measured before and after the experiment using the PANAS-SF (Positive and Negative Affect Schedule Short Form); ^9^ Trait and state anxiety were assessed by the STAI (State-Trait Anxiety Inventory) subtests X1 and X2 (maximum possible score 40 each). OXT, oxytocin; PLC, placebo.

**Table S2:** Overview of different constructs, definitions, items and sources

| **Construct** | **Definition** | **Sources** |
| --- | --- | --- |
| Sub-Construct | Items |  |
| **Commitment** | “An exchange partner believing that an ongoing relationship with another is so important as to warrant maximum efforts at maintaining it” (1) (p. 23) | 1 |
| Behavioral | The relationship with ... is something I am very committed to. | 1 |
|  | The relationship with ... is something I intend to maintain indefinitely. | 1 |
|  | I am willing to make sacrifices in order to maintain or engage in the relationship with ... . | 2 |
| Affective | I really like ... .^a^ | 2 |
|  | Thought of not being able to *have contact with/use* ... disturbs me. | 2 |
|  | No other *subject/brand* can take the place of ... . ^a^ | 3 |
| **Intimacy** | “Intimacy (…) refers to any close association or friendship that involves informal warmth, openness, and sharing" ^4^ (p. 127) | 5 |
|  | ... understands my needs. ^a^ | 2 |
|  | I know what ... stands for. | 2 |
|  | I would feel comfortable sharing detailed personal information about myself (like my address or phone number) with ... . | 5 |
|  | I would feel comfortable describing ... to someone who was not familiar with it. ^a^ | 5 |
| **Satisfaction** | A "cognitive and affective evaluation based on the personal experience across all (…) episodes within the relationship” ^6^ (p. 25) | 5 |
|  | ... takes care of me. | 2 |
|  | ... listens to me. ^a^ | 2 |
|  | I count on ... to do what’s best for me. ^a^ | 2 |
|  | ... is responsive to my concerns. | 2 |
| **Self-Connection** | “Reflects the degree to which (…) [a person or a] brand delivers on important identity concerns, tasks, or themes, thereby expressing a significant aspect of self” ^7^ (p. 364) | 5 |
|  | ... is part of me. ^a^ | 2 |
|  | ... successes are my successes. | 8 |
|  | I am interested in what others think about ... . | 8 |
|  | When someone criticizes ... , it feels like a personal insult. | 8 |
|  | If ... were disappeared from *my life/the market* I would be very distressed. ^a^ | 9 |
| **Trust** | “Trust is defined as a willingness to rely on an exchange partner in whom one has confidence” ^10^ (p. 82) | 10 |
|  | ... always keeps its promises. ^a^ | 11 |
|  | Whatever happens, I believe that I can rely on ... . ^a^ | 11 |
|  | I think ... cannot be trusted at times. | 1 |
|  | I think ... has high integrity. | 1 |
| **Loyalty** | "Maintenance of global trust - in a person, a party, an institution - even in circumstances where local disappointments might encourage its withdrawal” ^12^ (p. 218) |  |
| Cognitive | I believe ... provides more benefits than other/s *brands in its category*. ^a^ | 13 |
| Affective | I feel comfortable spending time with/using products of ... . | 13 |
| Conative | Even if *I get to know other interesting acquaintances/another brand is offering comparable products at a lower rate*, I’ll still *engage in an exchange with/buy from* ... . ^a^ | 13 |

*Notes.* Within the actual experiment subject or brand name was presented in place of “…”.^a^ Items used in the additional study with baseline salivary oxytocin concentrations.

**References**

1 Morgan, R. M. & Hunt, S. D. The Commitment-Trust Theory of Relationship Marketing. *J. Marketing* **58**, 20-38 (1994).

2 Fournier, S. Dimensionalizing brand relationships through brand relationship strength. Presentation at *Association for Consumer Research Conference* (2000).

3 Chang, P. L. & Chieng, M. H. Building consumer-brand relationship: A cross-cultural experiential view. *Psychol. Market.* **23**, 927-959 (2006).

4 Eshleman, J. R. & Clarke, J. N. *Intimacy, commitments, and marriage: Development of relationships* (Allyn and Bacon, New York, 1978).

5 Aaker, J., Fournier, S. & Brasel, S. A. When good brands do bad. *J. Consum. Res.* **31**, 1-16 (2004).

6 Storbacka, K., Strandvik, T. & Gronroos, C. Managing Customer Relationships for Profit - the Dynamics of Relationship Quality. *Int. J. Serv. Ind. Manag.* **5**, 21-38 (1994).

7 Fournier, S. Consumers and their brands: Developing relationship theory in consumer research. *J.Consum. Res.* **24**, 343-373 (1998).

8 Mael, F. & Ashforth, B. E. Alumni and Their Alma-Mater - a Partial Test of the Reformulated Model of Organizational Identification. *J. Organ. Behav.* **13**, 103-123 (1992).

9 Park, C. W., MacInnis, D. J., Priester, J., Eisingerich, A. B. & Lacobucci, D. Brand Attachment and Brand Attitude Strength: Conceptual and Empirical Differentiation of Two Critical Brand Equity Drivers. *J. Marketing* **74**, 1-17 (2010).

10 Moorman, C., Deshpande, R. & Zaltman, G. Factors Affecting Trust in Market-Research Relationships. *J. Marketing* **57**, 81-101 (1993).

11 Kim, H. K., Lee, M. & Lee, Y. W. *AP - Asia Pacific Advances in Consumer Research* [Ham Y.-U. & Duluth, Y. Y., (eds.)] [118-126] (Association for Consumer Research, 2005).

12 Gambetta, D. *Trust: Making and breaking cooperative relations* (Blackwell, Oxford, 1988).

13 Li, X. A. & Petrick, J. F. Reexamining the Dimensionality of Brand Loyalty: A Case of the Cruise Industry. *Journal of Travel & Tourism Marketing* **25**, 68-85 (2008).
